# Methylenetetrahydrofolate reductase polymorphisms and elevated plasma homocysteine levels in small vessel disease

**DOI:** 10.1002/brb3.2960

**Published:** 2023-03-28

**Authors:** Lei Chen, Chunhua Wu, Zhaoying Dong, Shanshan Cao, Ning Ren, Xiaoyan Yan

**Affiliations:** ^1^ Department of Neurology Tianjin Huanhu Hospital, Tianjin Key Laboratory of Cerebrovascular and Neurodegenerative Diseases Tianjin China; ^2^ Department of Neurology Tianjin Union Medical Center Tianjin China; ^3^ Department of Gerontology The No. 2 Hospital of Baoding Baoding China; ^4^ Peking University Clinical Research Institute Peking University First Hospital Beijing China

**Keywords:** homocysteine, methylenetetrahydrofolate reductase, small vessel disease

## Abstract

**Introduction:**

Despite its public health importance, the causes of small vessel disease (SVD) are not fully understood. The presence of SVD in monogenic twins indicates the involvement of genetic factors in the pathogenesis of this disease. The purpose of this study was to investigate the association of methylenetetrahydrofolate reductase (*MTHFR*) gene polymorphisms with SVD risk.

**Methods:**

Patients with SVD and matched controls were recruited from Tianjin Union Medical Center and Tianjin Huanhu Hospital. Clinical and laboratory data were collected. Plasma total homocysteine (tHcy) and folate levels were measured, and *MTHFR* rs1801133 (C677T) and rs1801131 (A1298C) single‐nucleotide polymorphisms were genotyped. We analyzed potential associations among SVD and *MTHFR* polymorphisms, tHcy, and folate levels.

**Results:**

Patients with SVD displayed significantly decreased plasma folate levels (*Z* = –3.537, *p* < .001) and increased tHcy levels (*Z* = 4.910, *p* < .001) compared with controls. Significantly different plasma tHcy levels were associated with rs1801133 (*χ* ^2^ = 6.664, *p* = .036), and post hoc analysis indicated higher plasma tHcy levels in individuals carrying the TT allele compared with levels in those carrying the TC allele (*Z* = 2.478, *p* = .013). No significant differences in tHcy levels were observed for rs1801131 alleles. The genotype and allele frequencies of rs1801133 were different between SVD and control groups (*χ* ^2^ = 9.378, *p* = .009). There was no significant difference in distributions of rs1801131 genotypes between the two groups, and multivariable logistic regression analysis showed that rs1801131 and rs1801133 were not significantly associated with the risk of SVD.

**Conclusions:**

Our study indicates that an elevated plasma tHcy level is independently associated with the development of SVD. Although *MTHFR* rs1801133 is linked to increased plasma homocysteine (Hcy) levels, it is not a risk factor for SVD. rs1801131 is not related to Hcy levels or SVD risk.

## Graphical abstract

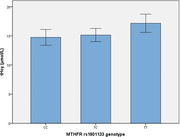

## 1 INTRODUCTION

Cerebral small vessel disease (SVD), a broad term encompassing a range of disease subtypes, accounts for approximately one fifth of strokes and is the major cause of vascular cognitive impairment and dementia (Markus & Erik de Leeuw, 2023). Radiological features include lacunar infarcts, white matter hyperintensities on T2/fluid‐attenuated inversion recovery magnetic resonance imaging (MRI), cerebral microbleeds, subcortical hemorrhage, and dilated perivascular spaces (Wardlaw et al., 2013). Although the pathological mechanisms of SVD remain incompletely understood, factors such as endothelial dysfunction, blood–brain barrier dysfunction, hypoperfusion, oxidative stress, and inflammation can contribute to its development (Ihara & Yamamoto, 2016; Low et al., 2019; Wardlaw et al., 2013). Among these factors, endothelial damage plays a crucial role during the entire disease pathogenesis (Quick et al., 2021; Rajani et al., 2018).

Homocysteine (Hcy) is a sulfur‐containing intermediary amino acid that is recycled by the remethylation pathway or converted into cysteine via the transsulfuration pathway (Moretti & Caruso, 2019; Smith & Refsum, 2016). Hcy accumulation can interfere with endothelial regulation, favor oxidative damage, and promote neuroinflammation and neurodegenerative processes (Hooshmand et al., 2016; Kloppenborg et al., 2014; Lee et al., 2019; Piao et al., 2018; Song et al., 2023). Endothelial dysfunction has been investigated as a major mechanism in the development of SVD (Li et al., 2021; Markus & Erik de Leeuw, 2023). Thus, an elevated total homocysteine (tHcy) level may be associated with various SVD components.

Methylenetetrahydrofolate reductase (MTHFR) is the rate‐limiting enzyme in Hcy metabolism, in which B vitamins folate, B12, and B6 are essential cofactors. Therefore, polymorphisms in *MTHFR* that affect its function can cause deficiencies in B vitamins that lead to increased blood levels of Hcy (Zaric et al., 2019). The homozygous form of *MTHFR* C677T reduces MTHFR activity and increases tHcy plasma concentrations; however, it remains uncertain whether this polymorphism is directly associated with SVD (Hassan et al., 2004; Rao et al., 2009; Zaric et al., 2019). Therefore, in the present study we investigated potential associations between susceptibility to SVD and the two most common single‐nucleotide polymorphisms (SNPs) in *MTHFR*: rs1801133 (C677T) and rs1801131 (A1298C).

## 2 METHODS

### 2.1 Participants

We recruited 200 SVD patients from outpatient and inpatient neurology clinics of Tianjin Union Medical Center and Tianjin Huanhu Hospital between September 2014 and May 2018. Included participants had a clinical lacunar stroke or probable transient ischemic attack with MRI evidence of a lacunar infarct, defined as a high‐signal lesion on diffusion‐weighted imaging or cavitated lacune of diameter ≤20 mm on T1‐weighted imaging, and confluent white matter hyperintensities of Fazekas grade ≥2. Exclusion criteria were as follows: less than 18 years of age; stroke mechanisms other than SVD, including cortical infarcts, cardioembolism, large vessel occlusion on magnetic resonance angiography or computed tomography angiography, other specific causes of stroke (e.g., arteritis, dissection), intra/extracranial large artery stenosis >50%, or subcortical infarct diameter >20 mm; history of major neurological or psychiatric condition except depression; malignancies; nutrient‐related disorders; or unsuitability for MRI. We recruited 200 age‐ and sex‐matched controls from the population who attended the hospital for annual physical examination. Control group participants had no medical history of a central nervous system ischemic event, major neurological disorders, psychiatric condition except depression, malignancies, or nutrient‐related disorders. The sample size was evaluated using an online sample‐size calculator (https://goodcalculators.com) before the study. The confidence level was set at 95% and the margin of error was set at 5%. The frequencies of rs1801133 and rs1801131 polymorphisms are approximately 20% and 31% according to a previous report, resulting in calculated sample sizes of 246 and 329, respectively. A value of 400 was determined to ensure statistical power.

### 2.2 Standard protocol approvals, registrations, and participant consent

The study was approved by the Ethics Committee of Tianjin Huanhu Hospital and conformed to the Declaration of Helsinki. The verbal consent obtained from participants or a legal representative was approved and the written informed consent was waived by the Ethics Committee of Tianjin Huanhu Hospital because the study did not include any therapeutic intervention or invasive procedures.

### 2.3 Clinical and laboratory evaluation

Demographic data and stroke risk factors were collected for both cohorts during face‐to‐face interviews and from hospital records. Fasting peripheral venous blood samples were collected from participants in the morning. Plasma fasting lipids and glucose were tested using automated clinical analyzers at the core laboratory of Tianjin Union Medical Center. Plasma folate, vitamin B12, and tHcy were measured using a chemiluminescent immunoassay (AxSYM; Abbott Laboratories, Abbott Park, IL, USA) within 2 h of collection at the same laboratory. The reference range for tHcy was 4.44–13.56 μmol/L. All diagnoses of diabetes mellitus were based on fasting glycemia ≥126 mg/dL (7 mmol/L) or hemoglobin A1c level ≥6.50%, and/or current antidiabetic treatment. Hypertension was defined as systolic and/or diastolic blood pressure of 140 and 90 mmHg, respectively (based on three independent measurements). Patients taking antihypertensive medications before stroke were considered hypertensive. Coronary artery disease (CAD) was assessed by electrocardiography and/or history of ischemic heart disease. Hyperlipidemia was diagnosed if (a) the patient had already taken statins or fenofibrate, (b) plasma cholesterol was ≥5.20 mmol/L, (c) triglyceride level was ≥1.70 mmol/L, or (d) low‐density lipoprotein level was ≥3.12 mmol/L. History of continuous smoking in the past or tobacco use at the time of stroke presentation was recorded. Current alcohol consumption was also included considering its influence on the methionine–Hcy cycle.

### 2.4 Genotyping

Peripheral blood samples (4 mL) of all participants were collected in 5.0‐mL EDTA tubes and stored at −80°C. Genomic DNA was extracted using a QIAamp DNA mini blood kit (Qiagen, Hilden, Germany) according to the manufacturer's instructions. *MTHFR* SNPs (rs1801133 and rs1801131) were genotyped using the commercially available Agena MassARRAY^®^ system (Agena Bioscience, San Diego, CA, USA) from Bio Miao Biological Technology (Beijing, China).

### 2.5 Statistical analysis

Differences in vascular risk factors (sex, age, hypertension, diabetes mellitus, CAD, and hyperlipidemia), lifestyle habits (smoking and drinking), plasma folate, and tHcy were compared between SVD and control groups using Student's *t* test or the Mann–Whitney *U* test (when a nonparametric test was required) for continuous variables and the *χ* ^2^ test for categorical variables. Associations of the two *MTHFR* SNPs with plasma folate and tHcy levels were tested using Mann–Whitney *U* and Kruskal–Wallis *H* tests. The level of statistical significance was set at *p* < .05. Variables with *p* < .05 in group comparison were entered into a logistic regression model to test the odds for SVD.

## 3 RESULTS

**FIGURE 1:**
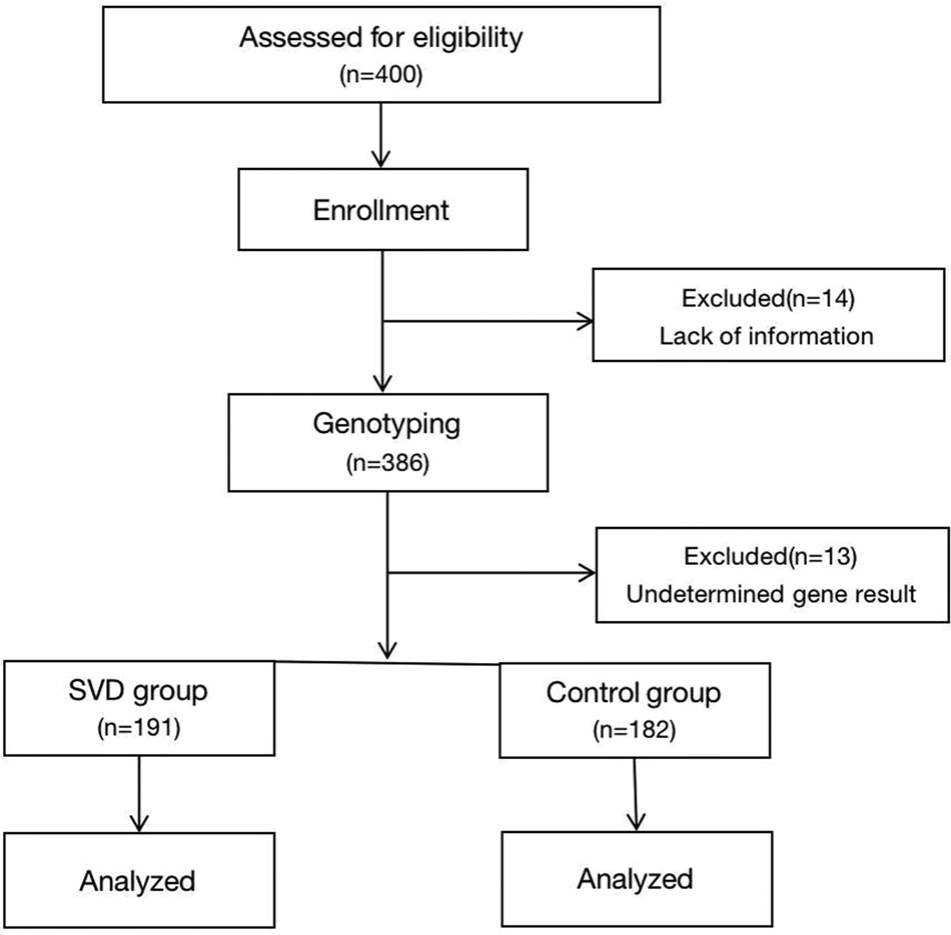
Flow chart of recruitment.

In total, 400 participants were initially included. Among these individuals, 14 participants were excluded because of incomplete information and the remaining 386 participants underwent blood testing for *MTHFR* genotyping. Of the 386 participants assessed, SNP detection rates were 99% for rs1801133 and 97% for rs1801131. An indeterminate genotype result was obtained for 13 individuals, who were therefore excluded, leaving 373 successfully genotyped subjects. A flow chart of recruitment is shown in Figure 1 and participant demographic data are summarized in Table 1. No significant differences attributable to age or sex were found between groups; however, the prevalence of hypertension, diabetes, CAD, hyperlipidemia, smoking, and drinking was higher in the SVD group compared with that in the control group. Patients with SVD had significantly lower plasma folate levels and significantly higher tHcy levels compared with controls (*Z* = −3.537 and 4.910, respectively, *p* < .001; Table 1).

**TABLE 1:** Characteristics of the study population

Variables	SVD (*n* = 191)	Control(*n* = 182)	*t*/*χ* ^2^/*Z*	*p*
Age (mean ± SD; years)	66.53 ± 8.82	66.77 ± 8.32	−0.271	.787
Sex, Male (%)	155 (81.15%)	152 (83.52%)	0.358	.550
HT, *n* (%)	171 (89.53%)	108 (59.34%)	45.056	<.001
DM, *n* (%)	77 (40.31%)	44 (24.18%)	11.075	.001
CAD, *n* (%)	72 (37.70%)	31 (17.03%)	19.907	<.001
HL, *n* (%)	56 (29.32%)	18 (9.99%)	22.122	<.001
Smoking, *n* (%)	118 (61.78%)	77 (42.31%)	14.164	<.001
Drinking, *n* (%)	81 (42.41%)	42 (23.08%)	15.758	<.001
Folate (ng/mL)	3.70 (2.80, 5.30)	4.30 (3.48, 6.42)	−3.537	<.001
tHcy (μmol/L)	15.30 (12.07, 20.00)	12.68 (12.26, 15.55)	4.910	<.001

*Note*: Variables are expressed as the mean ±standard deviation, median (interquartile range), or *n* (%).

Abbreviations: CAD, coronary artery disease; DM, diabetes mellitus; HL, hyperlipidemia; HT, hypertension; tHcy, total homocysteine.

The genotype and allele frequencies of rs1801131 and rs1801133 are presented in Table 2. rs1801131 C allele frequencies in SVD and control groups were 13.1% and 17.3%, respectively, while rs1801133 T allele frequencies were 61.8% and 51.7%, respectively. The genotype and allele frequencies of rs1801133 were significantly different between SVD and control groups (*χ* ^2^ = 9.378, *p* = .009). Specifically, there was a higher frequency of TT allele carriers in the SVD group compared with that in the control group. However, there was no significant difference in rs1801131 genotype distributions between the two groups.

**TABLE 2:** Genotype and allele frequencies of MTHFR rs1801131 and rs1801133 in SVD and control groups

SNP	Genotype	SVD, *n* (%)	Control	*χ* ^2^	*p*
rs1801131	AA	143 (74.9%)	122 (67.0%)	2.823	.244
	AC	46 (24.1%)	57 (31.3%)		
	CC	2 (1.0%)	3 (1.6%)		
rs1801133	CC	30 (15.7%)	40 (22.0%)	9.378	.009^*^
	TC	86 (45.0%)	97 (53.3%)		
	TT	75 (39.3%)	45 (24.7%)		

*Note*: Variables are expressed as *n* (%).

Abbreviation: SNP, single‐nucleotide polymorphism.

*p < .01.

**FIGURE 2:**
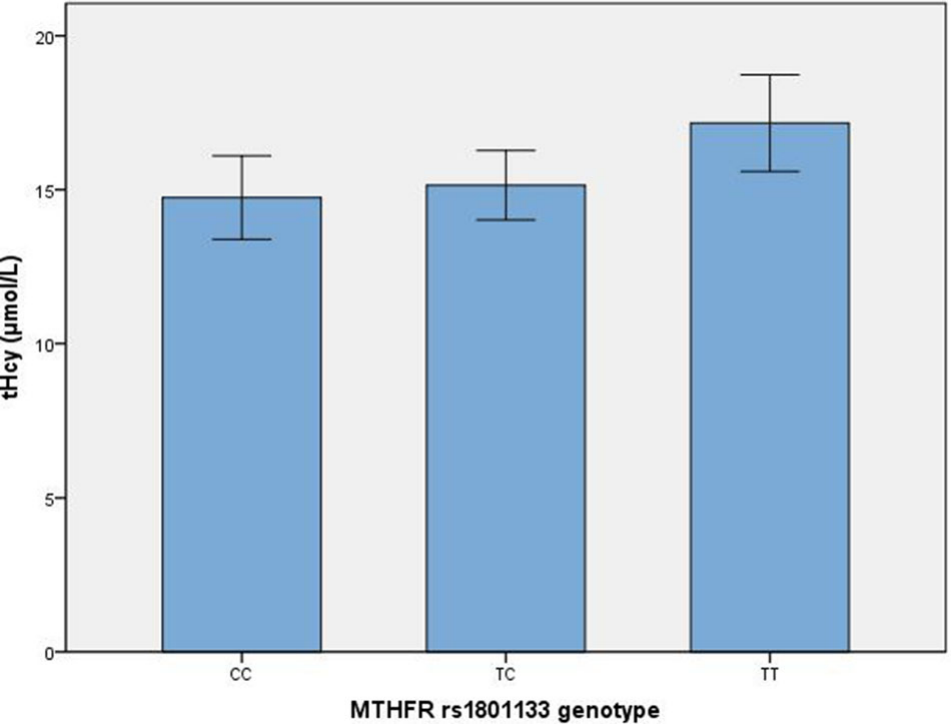
Comparison of plasma total homocysteine (Hcy) concentration for the MTHFR rs1801133 genotype showing individuals carrying the TT allele had higher levels of Hcy compared with those carrying the TC allele (*Z* = 2.478, *p* = .013).

Associations between folate and tHcy levels with *MTHFR* rs1801133 and rs1801131 are presented in Table 3 and Figure 2. There was no difference in drinking distribution among *MTHFR* rs1801131 (*χ* ^2^ = 2.213, *p* = .331) or rs1801133 (*χ* ^2^ = 0.698, *p* = .705) genotypes. However, significantly different plasma tHcy levels were associated with the *MTHFR* rs1801133 SNP (*F* = 6.664, *p* = .036). Post hoc analysis indicated higher plasma levels of tHcy in individuals carrying the TT allele compared with levels in those carrying the TC allele (*Z* = 2.478, *p* = .013), not CC allele (*Z* = 1.806, *p* = .071). No significant differences in tHcy levels were associated with rs1801131. No significant differences in folate levels were associated with rs1801131 or rs1801133 SNPs. In the *MTHFR* rs1801133 TT subgroup, patients with SVD had higher tHcy levels than controls (18.80 ± 8.26 vs. 14.44 ± 8.78 μmol/L, respectively; *t* = −2.732, *p* = .007), whereas folate levels were not significantly different between these two groups (4.87 ± 3.55 vs. 5.97 ± 3.53 μmol/L, respectively; *t* = 1.643, *p* = .103).

**TABLE 3:** Association of folate and tHcy levels with *MTHFR* rs1801133 and rs1801131

SNP (Genotype)		*N*	Folate (ng/mL)	*χ* ^2^	*p*	Hcy (μmol/L)	*χ* ^2^	*p*
rs1801131	AA	265	4.00 (3.00, 5.80)	0.540	.764	14.19 (11.18, 18.24)	1.789	.409
	AC	103	3.80 (3.20, 6.00)			13.27 (11.00, 17.32)		
	CC	5	4.50 (3.30, 8.55)			13.78 (13.46, 27.02)		
rs1801133	CC	70	3.80 (3.20, 6.43)	2.328	.312	13.46 (10.72, 16.55)	6.664	.036*
	TC	183	3.80 (3.10, 5.30)			13.25 (11.14, 16.98)		
	TT	120	4.40 (3.00, 6.38)			15.00 (11.64, 20.17)		

*Note*: Variables are expressed as n or the median (interquartile range).

Abbreviation: SNP, single‐nucleotide polymorphism.

*p < .05.

Multivariable logistic regression analysis was performed on candidate risk factors showing significant differences in group comparison. Hypertension, diabetes, CAD, hyperlipidemia, smoking, drinking, folate, Hcy, and the *MTHFR* SNP rs1801133 were selected to enter the regression model. Hypertension, diabetes, CAD, hyperlipidemia, drinking, and plasma Hcy level were independently associated with the risk of SVD; however, hyperhomocysteinemia only slightly contributed to the probability of SVD (odds ratio [OR] = 1.066, *p* < .001). Smoking and plasma folate were not significantly associated with the development of SVD. The OR of the rs1801133 TT allele was 2.002, but did not reach statistical significance. Details of the multivariable logistic regression analyses are shown in Table 4.

**TABLE 4:** Multivariable logistic regression analysis of SVD risk factors

Variables	*p*	OR	95% CI
HBP	<0.001**	6.146	3.163–11.943
DM	0.038*	1.763	1.033–3.010
CHD	<0.001**	2.778	1.583–4.874
HL	<0.001**	3.425	1.786–6.569
Smoking	0.471	1.233	0.698–2.176
Drinking	.001*	2.874	1.547–5.338
Folate	0.301	1.011	0.991–1.031
Hcy	<0.001**	1.066	1.029–1.105
rs1801133 CC	0.086	–	–
rs1801133 CT	0.703	1.138	0.584–2.216
rs1801133 TT	0.062	2.002	0.967–4.146

Abbreviations: CAD, coronary artery disease; DM, diabetes mellitus; HL, hyperlipidemia; HT, hypertension; OR, odds ratio; tHcy, total homocysteine.

*p < .05, **p < .001.

## 4 DISCUSSION

Common risk factors for SVD include hypertension, diabetes, and mutations in a number of genes (Choi, 2015; Hakim, 2019; Mok & Kim, 2015; Tan & Markus, 2015). The present study demonstrates that patients with SVD had a higher frequency of the *MTHFR* rs1801133 TT genotype, decreased folate, and increased tHcy plasma levels; however, rs1801131 had no association with folate or tHcy metabolism. Multivariable regression revealed independent associations of hypertension, diabetes, CAD, hyperlipidemia, drinking, and plasma Hcy level with the risk of SVD; however, folate and the rs1801133 TT genotype did not reach statistical significance as risk factors. Hypertension was proven to be the leading risk factor for the presence of SVD, while an elevated tHcy level rather than the *MTHFR* rs1801133 genotype had a weak effect on disease development. These findings indicate the need for Hcy‐lowering therapy and folate supplementation to prevent SVD in the aging population, not just individuals with the rs1801133 TT allele.

Although the pathogenesis of SVD is not fully understood, accumulating evidence indicates that an elevated tHcy level may be associated with various SVD components (Hooshmand et al., 2016; Jeon et al., 2014; Kloppenborg et al., 2014; Lee et al., 2019; Miwa et al., 2016; Moretti et al., 2021; Pavlovic et al., 2011; Song et al., 2023; Tan et al., 2017). In an extensive Chinese nationwide survey, 25.9% of Chinese people aged ≥40 years had hyperhomocysteinemia, which was independently associated with an increased risk of stroke in patients with hypertension (Tu et al., 2021). Another study of a healthy population showed that serum tHcy levels correlated with SVD development in a dose‐dependent manner (Nam et al., 2019). In a stroke prevention trial, patients with a low baseline Hcy level had better outcomes than those with a high baseline level (Spence et al., 2005). Our current findings are consistent with these previous studies. Therefore, the present study supports the idea that reducing Hcy levels is necessary for treatment of SVD considering the high prevalence of hyperhomocysteinemia, Hcy‐induced vulnerability, and limited time window for treatment.

Several factors, including genetic polymorphism, can affect Hcy metabolism. Numerous studies suggest that the *MTHFR* C677T variant increases the risk of mild tHcy elevation, while others observed no relationship between tHcy levels and C667T status in patients with acute ischemic stroke (Cardo et al., 2000; Castro et al., 2003). The lack of an association between tHcy levels and the *MTHFR* C677T genotype can be explained by genetics having less of an effect on tHcy compared with diet (Kluijtmans et al., 2003). Meanwhile, genetic association studies linking tHcy‐associated *MTHFR* variants to SVD have produced conflicting results. In a retrospective randomization analysis, rs1801133 was associated with folate and tHcy levels, and genetically higher tHcy levels were associated with greater risk of small vessel stroke (Larsson et al., 2019). In another study, *MTHFR* C677T was associated with lacunar stroke and white matter hyperintensity volume (Rutten‐Jacobs et al., 2016). However, in METASTROKE analysis, there was no association between *MTHFR* rs1801133 or rs1801131 SNPs and any form of ischemic stroke (Cotlarciuc et al., 2014). Given that our study failed to reveal a link between the *MTHFR* variant and SVD, an Hcy‐targeted intervention for SVD prevention and treatment does not need to rely on the individual's genotype.

Association of rs1801131 with tHcy and stroke susceptibility is inconsistent. In a meta‐analysis including 5725 cases and 8655 controls, evidence supported a correlation between *MTHFR* rs1801131 and stroke susceptibility (Dong et al., 2021). However, few studies have focused on rs1801131 and SVD; indeed, only one study has shown an association between mutations at A1298C with decreased tHcy levels and reduced brain parenchymal fractions (Cao et al., 2021). We detected no relationship between rs1801131 and tHcy levels or increased SVD risk. A1298C is associated with a decrease in *MTHFR* activity but not increased Hcy plasma concentrations (Weisberg et al., 1998). Functional differences between these two SNPs can be explained by their location: C677T is located within the N‐terminal catalytic region, while A1298C is located within the C‐terminal regulatory domain involved in enzyme regulation.

Limitations of the present study include a relatively small population, which failed to provide sufficient power for statistical significance. Furthermore, a high percentage of male participants in the study population may bias outcomes, although the case and control groups were well balanced. Finally, the participants’ diet was not taken into consideration despite its effects on Hcy levels.

In conclusion, the findings of this study indicate that an elevated plasma tHcy level is independently associated with the development of SVD. Although the *MTHFR* rs1801133 polymorphism is linked to an elevated plasma Hcy level, it is not a risk factor for SVD. The *MTHFR* rs1801131 polymorphism is not related to Hcy levels or SVD risk. However, given the inconsistent results of *MTHFR* polymorphism studies, further explorations are needed to confirm the role of these variants in SVD.

## AUTHOR CONTRIBUTIONS

Lei Chen drafted and oversaw implementation of the study and drafted the manuscript. Chunhua Wu, Zhaoying Dong, Shanshan Cao, and Ning Ren supported recruitment and acquired data. Xiaoyan Yan analyzed the data. All authors read and approved the final manuscript.

## CONFLICT OF INTEREST STATEMENT

The authors declare no conflicts of interest.

### PEER REVIEW

The peer review history for this article is available at https://publons.com/publon/10.1002/brb3.2960.

## Data Availability

The original contributions presented in the study are included in the article; further inquiries can be directed to the corresponding author.
